# Pregnancy-Related Systemic Lupus Erythematosus: Clinical Features, Outcome and Risk Factors of Disease Flares — A Case Control Study

**DOI:** 10.1371/journal.pone.0104375

**Published:** 2014-08-13

**Authors:** Huaxia Yang, Hui Liu, Dong Xu, Lidan Zhao, Qian Wang, Xiaomei Leng, Wenjie Zheng, Fengchun Zhang, Fulin Tang, Xuan Zhang

**Affiliations:** 1 Department of Rheumatology, Peking Union Medical College Hospital, Peking Union Medical College, Chinese Academy of Medical Sciences, Beijing, China; 2 Department of Rheumatology, Dongfang Hospital, The Second Clinical Medical College of Beijing University of Chinese Medicine, Beijing, China; Keio University School of Medicine, Japan

## Abstract

**Objective:**

To investigate the clinical features, outcome, and risk factors of disease flares in patients with pregnancy-related lupus (PRL).

**Methods:**

Medical charts of 155 consecutive PRL inpatients were systematically reviewed, including demographic data, clinical features, laboratory findings, treatment, complications, and outcome.

**Results:**

PRL cases were divided into active (a-PRL) (n = 82, 53.0%) and stable lupus (s-PRL) (n = 73, 47.0%). Compared with nonpregnant active female systemic lupus erythematosus (SLE) patients, a-PRL including new-onset lupus (n-PRL) and flare lupus (f-PRL) (n = 41 respectively), had a higher incidence of renal and hematological involvement but less mucocutaneous and musculoskeletal involvement (p<0.05). The incidence of preeclampsia/eclampsia, fetal loss, and preterm birth were significantly higher in a-PRL than in s-PRL (p<0.05). Despite receiving a more vigorous glucocorticoid treatment, a-PRL mothers had a poorer prognosis (p<0.001). Five (6.1%) of them died and 13 (15.9%) developed severe irreversible organ failure, whereas none of these events was observed in the s-PRL group. Multivariate logistic analysis indicated that a history of lupus flares and serological activity (hypocomplementemia and/or anti-dsDNA positivity) at the time of conception were associated with lupus flares in PRL mothers.

**Conclusions:**

SLE patients with a flare history and serological activity at the time of conception were at an increased risk of disease flares during pregnancy and puerperium. a-PRL patients were more prone to renal and hematological involvement, pregnancy complications, and a poorer prognosis despite more vigorous glucocorticoid treatment.

## Introduction

Systemic lupus erythematosus (SLE) is a severe rheumatic disease that usually affects women of childbearing age. In fact, some patients incur this disorder during pregnancy and puerperium, whereas in established SLE, the disease is inclined to exacerbate [1]–[3]. The recognition of lupus exacerbation is sometimes difficult because the clinical symptoms may mimic those related to pregnancy. Moreover, the prompt management of lupus in the mother and at the same time appropriate maintenance of normal fetal development poses a great challenge to clinicians. So far, there have been a few reports on maternal and fetal complications in pregnancy-related lupus (PRL) [4]–[10]. However, most of them are studies with small sample sizes and a detailed study of the clinical features of PRL is lacking. In addition, the factors predicting lupus flares remain unexplored. In this study, we examined the medical charts of all PRL inpatients admitted in the past 20 years to Peking Union Medical College Hospital (PUMCH), the tertiary referral center in China, and assessed the clinical characteristics and disease/pregnancy outcomes; furthermore, we investigated the risk factors of disease flares in PRL patients.

## Methods

### 1. Patients and controls

We collected data on 155 consecutive PRL inpatients who were admitted to PUMCH from 1992 to 2012. Among 4,456 nonpregnant active lupus inpatients treated during the same period, we randomly selected 164 age-matched female patients as controls. The medical charts of all these patients were systematically reviewed by three rheumatologists (HY, HL, and DX), including demographic data, clinical manifestations, lab findings, treatment, and prognosis. All the PRL patients were followed up for 6 months after the termination of pregnancy. This study was approved by the Ethics Committee of PUMCH. This was a retrospective study, collecting data during the past 20 years. The patients enrolled in this study during the most recent 2 to 3 years had provided their written informed consent, and the remaining were contacted by phone and provided their verbal informed consent. When we got informed consent, all the patients were at childbearing age and no children were enrolled. This consent procedure was approved by the Ethics Committee of PUMCH.

### 2. Classification of SLE and PRL

SLE patients were diagnosed according to the 1997 revised classification criteria of the American College of Rheumatology. The disease activity score was calculated using the SLE disease activity index (SLEDAI) [11]. PRL patients were classified into three groups: 1) Flare of established lupus (f-PRL): patients had active lupus with SLEDAI>4 and at least one of the organs (renal, cardiovascular, respiratory, nervous, gastrointestinal, or hematological system) was affected during pregnancy and puerperium. These patients were at stable disease status (SLEDAI≤4) just before the conception. 2) Stable lupus (s-PRL): SLEDAI≤4, with no clinical manifestation suggesting the involvement of any of the above organs during pregnancy and puerperium. 3) New-onset lupus (n-PRL): SLE newly occurred during the pregnancy and puerperium.

### 3. Statistical analysis

The Statistical Package for the Social Sciences (SPSS), version 17.0 (SPSS Inc., Chicago, IL, USA) was used for data analysis. Descriptive data are depicted as mean ± standard deviation, median and interquartile range, or frequency (percentage). Statistical analysis included the chi-squared test, Fisher's exact test, Mann-Whitney U-test, and Student's t-test as appropriate. The risk factors for lupus flares during pregnancy were analyzed using binary logistic regression analysis. Entry and removal probabilities for stepwise regression were 0.05 and 0.1, respectively. In all tests, the probability values were two-sided, and p values <0.05 were considered significant.

## Results

### 1. Clinical features

#### 1.1 Demographic data

Among the 155 PRL patients, 41 (26.5%), 41 (26.5%), and 73 (47.0%) cases were n-PRL, f-PRL, and s-PRL, respectively. There were no significant differences in age at disease onset and the percentage of the first conception among the three PRL groups and in disease duration between f-PRL and s-PRL (p>0.05) (Table 1). All n-PRL cases had active lupus with SLEDAI>4, and therefore more than half (n = 82, 53.0%) of the cases in this cohort of PRL had active disease. Among the 82 a-PRL patients (n-PRL+f-PRL), 26 (31.7%), 26 (31.7%), 24 (29.3%), and 6 (7.3%) cases incurred active disease during their first, second, and third trimesters and puerperium, respectively.

**Table 1:** Demographic features.

	n-PRL (n = 41)	f-PRL (n = 41)	s-PRL (n = 73)
Age at onset (years)	25.61±4.34	21.51±5.06	23.01±5.34
Disease duration (months)	NA	82.76±41.95	72.19±32.87
SLEDAI			
Maximum during pregnancy and puerperium	11.51±6.92	9.32±4.23	0.76±0.74
Just before conception	NA	0,3	0, 0
The first conception	15 (36.6%)	18 (43.9%)	31 (42.4%)

Data are depicted as mean ± standard deviation; median, interquartile range; or number (%).

n-PRL: new-onset lupus during pregnancy and puerperium.

f-PRL: flare of established lupus during pregnancy and puerperium.

s-PRL: established lupus remained stable during pregnancy and puerperium.

SLEDAI: systemic lupus erythematosus disease activity index.

NA: not available.

#### 1.2 Clinical manifestations

We compared a-PRL patients with nonpregnant active female SLE patients admitted to PUMCH during the same time period (n = 164). As shown in Table 2, a-PRL patients had a higher incidence of renal and hematological involvement as well as thrombotic thrombocytopenic purpura (TTP) but less mucocutaneous and musculoskeletal involvement than nonpregnant active SLE patients (p<0.05). In addition, n-PRL patients had a higher incidence of interstitial lung disease than f-PRL and nonpregnant active SLE patients (p<0.05). f-PRL patients had a higher incidence of renal involvement but a lower incidence of musculoskeletal involvement than n-PRL and nonpregnant active SLE patients (p<0.05). Though PRL patients may develop active disease during all three trimesters, there was no significant difference of clinical features of lupus flare among patients in different trimesters (p>0.05).

**Table 2:** Clinical manifestations: a-PRL vs. Nonpregnant active SLE.

Clinical manifestations	a-PRL			Nonpregnant active SLE (n = 164)
	n-PRL (n = 41)	f-PRL (n = 41)	Total (n = 82)	
Mucocutaneous	20 (48.8%)	15 (36.6%)#	35 (42.7%)*	98 (59.8%)
Facial rash	16 (39.0%)	9 (22.0%)#	25 (30.5%)*	77 (47.0%)
Oral ulcer	6 (14.6%)	1 (1.4%)#	7 (8.5%)	19 (11.6%)
Alopecia	8 (19.5%)	6 (14.6%)	14 (17.1%)	45 (27.4%)
Photosensitivity	5 (12.2%)	5 (12.2%)	10 (12.2%)	19 (11.6%)
Musculoskeletal	14 (34.1%)	3 (7.3%)# Δ	17 (20.7%)*	56 (34.1%)
Arthritis	12 (29.3%)	3 (7.3%)# Δ	15 (18.3%)*	52 (31.7%)
Myositis	3 (7.3%)	0 (0.0%)	3 (3.7%)	9 (5.5%)
Raynaud's phenomenon	6 (14.6%)	4 (9.8%)	10(12.2%)	31(18.9%)
Renal	27 (65.9%)	35 (85.4%)# Δ	62 (75.6%)*	102 (62.2%)
Proteinuria	23 (56.1%)	36 (90.0%)# Δ	59 (72.8%)*	92 (56.1%)
Hematuria	19 (46.3%)	22 (55.0%)#	41 (50.0%)*	65 (39.6%)
Nephritic syndrome	10 (24.4%)	19 (46.3%)	29 (35.4%)	58 (35.4%)
Renal insufficiency (Scr >132.6 mmol/L)	2 (4.9%)$	3 (7.5%)	5 (6.2%)*	26 (15.9%)
Cardiovascular	8 (19.5%)	9 (22.0%)	17 (20.7%)	48 (29.3%)
Pulmonary artery hypertension	8 (19.5%)	4 (9.8%)	12 (14.6%)	23 (14.2%)
Pulmonary	9 (22.0%)Δ	2 (4.9%)	11 (13.4%)	26 (15.9%)
Interstitial lung disease	8 (19.5%)$ Δ	1 (2.4%)	9 (11.0%)*	4 (2.4%)
Alveolar hemorrhage	2 (4.9%)	1 (2.4%)	3/79 (3.7%)	4 (2.4%)
Nervous system	7 (17.1%)	6 (15.0%)	13 (16.0%)	40 (24.4%)
central	6 (14.6%)	6 (15.0%)	12 (14.8%)	35 (21.3%)
peripheral	1 (2.4%)	0 (0.0%)	1 (1.2%)	6 (3.7%)
Gastrointestinal	10 (24.4%)	10 (24.4%)	20 (24.4%)	30 (18.5%)
Hematological	25 (61.0%)$	23 (56.1%)	48 (58.5%)*	71 (43.5%)
Hemolytic anemia	1 (2.4%)	2 (5.0%)	3 (3.7%)	7 (4.3%)
Leukocyte <4×10^9^/L	9 (22.0%)	4 (9.8%)#	13 (15.9%)*	54 (32.9%)
Platelet <100×10^9^/L	16 (40.0%)	22 (53.7%)#	38 (46.9%)*	43 (26.2%)
Thrombotic thrombocytopenic purpura	5 (12.2%)$	1 (2.4%)	6 (7.3%)*	0 (0%)
Antiphospholipid syndrome	8 (19.5%)	6 (14.6%)	14 (17.1%)	17 (10.4%)

Data are depicted as number (%).

PRL: pregnancy-related lupus.

SLE: systemic lupus erythematosus.

a-PRL: active lupus during pregnancy and puerperium.

n-PRL: new-onset lupus during pregnancy and puerperium.

f-PRL: flare of established lupus during pregnancy and puerperium.

$ : n-PRL vs. Nonpregnant active SLE, p<0.05.

# : f-PRL vs. Nonpregnant active SLE, p<0.05.

*: Total of active PRL vs. Nonpregnant active SLE, p<0.05.

Δ : n-PRL vs. f-PRL, p<0.05.

#### 1.3 Laboratory Results

There was no significant difference between a-PRL and nonpregnant active SLE patients in terms of the levels of erythrocyte sedimentation rate (ESR) or complements (C3, C4, CH50), or the proportion of patients with positive anti-dsDNA, anti-SSA/anti-Ro, anti-SSB/anti-La, anti-ribosomal P, anti-U1RNP, anti-Sm, anticardiolipin (ACL), or anti-beta-2 glycoprotein 1(anti-β2GP1) antibodies (p>0.05). More n-PRL patients had elevated IgG than f-PRL and nonpregnant active SLE patients (p<0.05) (Table 3).

**Table 3:** Laboratory findings: Active PRL vs. Nonpregnant active SLE.

Laboratory findings	a-PRL			Nonpregnant active SLE (n = 184)
	n-PRL (n = 41)	f-PRL (n = 41)	Total (n = 82)	
ESR (mm/h)	62.21±32.98	47.65±27.14	54.73±30.80	47.96±18.95
C3 (g/L)	0.69±0.39	0.65±0.24	0.67±0.32	0.58±0.31
C4 (g/L)	0.10, 0.09	0.11, 0.08	0.10, 0.08	0.11, 0.10
CH50 (g/L)	36.70±22.26	39.92±18.45	38.46±20.13	33.26±21.48
IgG elevation	25 (60.9%)$ Δ	4 (9.8%)	29 (35.4%)	62 (33.7%)
IgA elevation	4 (9.8%)	8 (19.5%)	12 (14.6%)	39 (21.2%)
IgM elevation	0 (%)	0 (0%)	0 (0%)	13 (7.1%)
Anti-dsDNA (+)	18 (43.9%)	19 (46.3%)	37 (45.1%)	75 (40.8%)
Anti-SSA/Anti-Ro (+)	23 (56.1%)	24 (58.5%)	47 (57.3%)	81 (44.0%)
Anti-SSB/Anti-La (+)	6 (14.6%)	3 (7.3%)	9 (11.0%)	15 (8.2%)
Anti-ribosomal P (+)	4 (9.8%)	4 (9.4%)	8 (9.8%)	28 (15.2%)
Anti-U1RNP (+)	18 (43.9%)	14 (34.1%)	32 (39.0%)	53 (28.8%)
Anti-Sm (+)	8 (19.5%)	10 (24.4%)	18 (22.0%)	32 (17.4%)
ACL/Anti-β2GP1 (+)	6 (14.6%)	7 (17.0%)	13 (15.9%)	20 (10.9%)

Data are depicted as mean ± standard deviation; median, interquartile range; or number (%).

PRL: pregnancy-related lupus.

SLE: systemic lupus erythematosus.

a-PRL: active lupus during pregnancy and puerperium.

n-PRL: new-onset lupus during pregnancy and puerperium.

f-PRL: flare of established lupus during pregnancy and puerperium.

ESR: erythrocyte sedimentation rate.

ACL: anticardiolipin antibody.

$ : n-PRL vs. Nonpregnant active SLE, p<0.05.

Δ : n-PRL vs. f-PRL, p<0.05.

### 2. Pregnancy complications, treatment, and prognosis

All PRL patients were followed up for 6 months after the termination of their pregnancy. As shown in Table 4, compared with the s-PRL patients, the incidences of preeclampsia/eclampsia, fetal loss, and preterm birth were significantly higher in the n-PRL and f-PRL groups (p<0.05). However, the incidence of induced abortion was significantly higher in the s-PRL group (p<0.05). Despite receiving a more vigorous glucocorticoid treatment, a-PRL mothers had a poorer prognosis (p<0.001). Of the 82 a-PRL patients, 5 (6.1%) died and 13 (15.9%) developed severe irreversible organ failure whereas none of these events was observed in the s-PRL group. The causes of death for each of the patients were lupus cerebritis, TTP-complicated alveolar hemorrhage, septic shock, acute pulmonary embolus, and cardiac sudden death associated with pulmonary artery hypertension, respectively.

**Table 4:** Pregnancy complications, treatment, and prognosis: Active PRL vs. s-PRL.

Complications, treatment, and prognosis	a-PRL			s-PRL (n = 73)
	n-PRL (n = 41)	f-PRL (n = 41)	Total (n = 82)	
Preeclampsia/eclampsia	6 (14.6%)	20 (48.8%)# Δ	26 (31.7%)*	7 (9.6%)
Fetal loss	16 (39.0%)$	13 (31.7%)#	29 (35.4%)*	8 (11.0%)
Spontaneous abortion	13 (31.7%)$	10 (24.4%)#	23 (28.0%)*	7 (9.6%)
Prenatal death	3 (7.3%)	3 (7.3%)	6 (7.3%)	1 (1.4%)
Stillbirth	1 (2.4%)	2 (4.9%)	3 (3.6%)	1 (1.4%)
Neonatal death	2 (4.9%)	1 (2.4%)	3 (3.6%)	0 (0.0%)
Induced abortion	5 (12.2%)$	6 (14.6%)#	11 (13.4%)*	23 (31.5%)
Live birth	20 (48.8%)	22 (53.7%)	42 (51.2%)	42 (57.5%)
Preterm birth	15 (36.6%)$	17 (41.5%)#	32 (39.0%)*	4 (5.5%)
Glucocorticoid (methylprednisolone pulse/ 1–2 mg.kg-^1^ d^−1^prednisone/<1 mg.kg^−1^ d^−1^ prednisone)	10/20/11$	7/28/6#	17/48/17*	0/0/73
Maternal death/severe irreversible organ failure	10 (24.4%)$	8 (19.5%)#	18 (21.9%)*	0 (0%)
Maternal death	2 (4.9%)	3 (7.3%)	5 (6.1%)*	0 (0%)
Maternal severe irreversible organ failure	8 (19.5%)$	5 (12.2%)#	13 (15.9)*	0 (0%)

Data are depicted as n (%).

PRL: pregnancy-related lupus.

a-PRL: active lupus during pregnancy and puerperium.

n-PRL: new-onset lupus during pregnancy and puerperium.

f-PRL: flare of established lupus during pregnancy and puerperium.

s-PRL: established lupus remained stable during pregnancy and puerperium.

Spontaneous abortion: spontaneous fetal loss before 28 weeks of gestation.

Stillbirth: intrauterine fetal demise after 28 weeks of gestation.

Neonatal death: live infant dying within 28 days after delivery.

Induced abortion: voluntarily induced termination of pregnancy.

Severe irreversible organ failure includes: (1) serum creatinine ≥442 mmol/L leading to lifetime hemodialysis or kidney transplantation; and/or (2) pulmonary artery hypertension with heart failure; and/or (3) intestinal pseudo-obstruction leading to lifetime parenteral nutrition.

$ : n-PRL vs. s-PRL, p<0.05.

# : f-PRL vs. s-PRL, p<0.05.

*: Total of active PRL vs. s-PRL, p<0.05.

Δ : n-PRL vs. f-PRL, p<0.05.

### 3. Risk factors for SLE flares during pregnancy and puerperium

To evaluate the risk factors of lupus flares during pregnancy and puerperium, we extracted the baseline data at the time of conception, including demographic data, past medical history, and clinical and laboratory findings; the data were compared between the f-PRL and s-PRL groups (Table 5). By single factor analysis, statistically significant differences between the f-PRL and s-PRL groups were found for the following variables: history of lupus flares, proteinuria, and serological activity (hypocomplementemia and/or anti-dsDNA positivity) at the time of conception. These variables were further analyzed with the logistic regression model, and the serological activity at the time of conception and the flare history were found to be risk factors for lupus flares during pregnancy and puerperium (Table 6).

**Table 5:** Comparison of baseline variables between f-PRL and s-PRL at the time of conception.

Variables	f-PRL (n = 41)	s-PRL (n = 73)	P value
Age at diagnosis (years)	21.55±5.08	22.99±5.34	0.167
Duration of SLE (months)	82.76±41.95	72.19±32.87	0.215
Time lag between onset and initial treatment (months)	3, 9	3, 6	0.931
History of renal involvement	20 (50.0%)	25 (34.7%)	0.159
History of musculoskeletal involvement	15 (37.5%)	25 (34.2%)	0.837
History of skin involvement	18 (45.0%)	27 (37.0%)	0.428
History of lupus flares	17 (45.9%)	7 (9.7%)	<0.001
Cumulative glucocorticoid past dose (g)	16.3, 27.9	10.9, 15.0	0.107
Proteinuria	7 (17.9%)	2 (2.8%)	0.009
Leukocyte <4×10^9^/L	1 (2.6%)	1 (1.4%)	0.680
Platelet <100×10^9^/L	3 (7.7%)	3 (4.3%)	0.466
Serological activity	12 (29.3%)	2 (2.7%)	<0.001
Hypocomplementemia	10 (25.0%)	1 (1.4%)	<0.001
Anti-ds DNA (+)	10 (25.6%)	2 (2.8%)	<0.001
Anti-SSA/Anti-Ro (+)	19 (59.4%)	19 (54.3%)	0.806
Anti-SSB/Anti-La (+)	2 (6.3%)	5 (14.3%)	0.431
Anti-ribosomal P (+)	1 (3.1%)	1 (2.7%)	0.485
Anti-U1RNP (+)	11 (34.4%)	8 (23.6%)	0.475
ACL/anti-β2GP1 (+)	5 (16.7%)	2 (9.1%)	0.685

Data are depicted as mean ± standard deviation; median, interquartile range; or number (%).

PRL: pregnancy-related lupus.

f-PRL: flare of established lupus during pregnancy and puerperium.

s-PRL: established lupus remained stable during pregnancy and puerperium.

ACL: anticardiolipin antibody.

**Table 6:** Binary logistic regression analysis of risk factors for disease flares in SLE patients during pregnancy.

Variable	Coefficient	S.E.	Wald Chi-square	P value	OR	95% C.I. for OR
Serological activity at the time of conception	0.837	0.394	4.510	0.034	5.341	1.138–25.067
History of SLE flares	0.810	0.282	8.253	0.004	5.061	1.674–15.298
Proteinuria at the time of conception	0.135	0.596	0.051	0.821	1.309	0.126–13.553

## Discussion

The appropriate management of SLE patients during pregnancy is a challenge to both rheumatologists and obstetricians. Pregnancy may trigger lupus flares, whereas SLE can also lead to an unfavorable pregnancy outcome. A previous study has suggested that to achieve an optimal prognosis for both the mother and fetus, SLE patients should have quiescent disease for at least 6 months prior to conception. However, the rate of lupus exacerbation still ranges from 7% to 33%. If SLE patients had active disease at the time of conception, the rate could reach 60% [12].

In this study, we systematically reviewed all of the consecutive PRL patients admitted to PUMCH in the past 20 years. We found that more than half of the PRL patients had active disease during pregnancy and puerperium. Importantly, n-PRL patients comprised half of the a-PRL patients and had distinctive clinical features including more interstitial lung disease and TTP than the other PRL and nonpregnant SLE patients. This observation has been rarely reported previously and is notable [2]. It is interesting that anti-U1RNP was somewhat high in n-PRL, which could be a possible explanation of the higher complication of ILD in these patients. Though there have been reports suggesting that the frequency of disease flares may decrease in the third trimester [13], [14], this study demonstrated that PRL patients may develop active disease during all three trimesters with almost equal frequency.

In contrast with the nonpregnant active SLE patients, the a-PRL patients had more proteinuria/hematuria and thrombocytopenia but less skin and joint manifestations, suggesting that pregnancy may be associated with more severe organ involvement. Intriguingly, a higher incidence of TTP was observed in the n-PRL group, which may be related to the increased platelet consumption in pregnancy and microangiopathies. Different types of microangiopathies can occur during pregnancy, including hemolysis-elevated liver enzymes-low platelets syndrome, eclampsia or preeclampsia, TTP, and antiphospholipid syndrome, leading to thrombocytopenia in pregnant women [15]. The prompt differential diagnosis of these pathological conditions is critical because their managements may vary depending on the type of microangiopathy. Four of the six PRL-TTP mothers in this cohort had a poor prognosis despite receiving plasma exchange as well as bolus methylprednisolone therapy. One case died and three had renal failure. But in general, though more a-PRL patients had proteinuria/hematuria than active nonpregnant active SLE patients, the frequency of renal insufficiency was significantly lower in a-PRL patients. The major reason might be that patients with established lupus and existing severe kidney damage were usually advised against childbirth by their physicians.

Reports on lupus prognosis of PRL patients are rare. Our study observed that a-PRL patients had a poorer maternal prognosis, despite more vigorous treatment with glucocorticoids and/or immunosuppressants. All 5 deaths and 13 cases of severe irreversible organ failure occurred in the a-PRL group. In addition, the a-PRL patients had significantly higher incidences of preeclampsia/ecalmpsia, fetal loss, and preterm birth than the s-PRL patients. However, in the s-PRL patient group, the rate of induced abortion was unexpectedly high, reflecting limited access to a qualified rheumatologist and the lack of collaboration between the rheumatologist and obstetrician [16], [17].

This study also reported that the risk factors for patients developing active disease during pregnancy and puerperium included a history of lupus flares as well as serological activity at the time of conception. These results highlight the importance of close monitoring of disease activity during pregnancy in SLE patients with serological activity despite having clinically quiescent disease at the time of conception, in which case the change of maternal hormonal levels and the increased estrogen level may trigger disease flares during pregnancy [18].

This study also has limitations. As it was a retrospective study, we didn't follow up this cohort of patients for long-term prognosis. Future multi-centre prospective studies are needed to confirm our findings. In conclusion, this study revealed that SLE patients with a flare history and serological activity at the time of conception were at an increased risk of disease flares during pregnancy and puerperium. Therefore it should be prudent for SLE patients with a flare history and are still serological active to consider gestation. It is clinically important to discriminate a-PRL patients, as they were more prone to renal and hematological involvement, pregnancy complications, and a poorer prognosis despite more vigorous glucocorticoid treatment.
